# The impact of food insecurity on HIV outcomes in Senegal, West Africa: a prospective longitudinal study

**DOI:** 10.1186/s12889-021-10444-1

**Published:** 2021-03-06

**Authors:** Noelle A. Benzekri, Jacques F. Sambou, Sanou Ndong, Mouhamadou Baïla Diallo, Ibrahima Tito Tamba, Dominique Faye, Ibrahima Sall, Jean Philippe Diatta, Khadim Faye, Ousseynou Cisse, Fatima Sall, Ndèye Fatou Ngom Guèye, Cheikh T. Ndour, Papa Salif Sow, Jean Jacques Malomar, Stephen E. Hawes, Moussa Seydi, Geoffrey S. Gottlieb

**Affiliations:** 1 Department of Medicine, University of Washington, Box 358061, 750 Republican St., Seattle, WA 98109-4725 USA; 2 Centre de Santé de Ziguinchor, Ziguinchor, Senegal; 3 Services des Maladies Infectieuses et Tropicales, Centre Hospitalier National Universitaire de Fann, Dakar, Senegal; 4 Centre de Santé de Bignona, Bignona, Senegal; 5 Division de Lutte contre le Sida et les IST, Ministère de la Santé et de l’Action Sociale, Dakar, Senegal; 6 Department of Epidemiology, University of Washington, Seattle, WA USA; 7 Department of Global Health, University of Washington, Seattle, WA USA

**Keywords:** HIV/AIDS, Food insecurity, Virologic failure, Loss to follow-up, Adherence, Nutrition, Stigma, Social determinants, Agriculture, Care cascade, Senegal, West Africa

## Abstract

**Background:**

Understanding the impact of food insecurity on HIV outcomes is critical for the development and implementation of effective, evidence-based interventions to address food insecurity and improve the HIV care cascade. We conducted a prospective, longitudinal study to determine the impact of food insecurity on HIV outcomes in Senegal, West Africa.

**Methods:**

HIV-infected individuals presenting for care and initiation of ART through the Senegalese National AIDS program in Dakar and Ziguinchor were eligible for enrollment. Data were collected using interviews, clinical evaluations, laboratory analyses, and chart review at enrollment, month 6, and month 12. Logistic regression was used to determine the association between food insecurity and HIV outcomes.

**Results:**

Among the 207 participants in this study, 70% were female and the median age was 37 years. The majority (69%) were food insecure at enrollment, 29% were severely food insecure, and 38% were undernourished. Nearly a third (32%) had no formal education, 23% practiced agriculture, and 40% owned livestock. The median daily food expenditure per person was $0.58. The median round trip transportation time to clinic was 90 min (IQR 30–240). The median cost of transportation to clinic was $1.74. At month 12, 69% were food insecure, 23% were severely food insecure, and 14% were undernourished. At month 12, 43% had not disclosed their HIV status; food insecurity was associated with non-disclosure of HIV-status due to fear of stigmatization and feelings of shame. Severe food insecurity was a strong predictor of loss to follow-up (OR 3.13 [1.08–9.06]) and persistent severe food insecurity was associated with virologic failure (OR 5.14 [1.01–26.29]) and poor adherence to ART 8.00 [1.11–57.57]. Poor nutritional status was associated with poor immunologic recovery (OR 4.24 [1.56–11.47]), virologic failure (OR 3.39 [1.13–10.21]), and death (OR 3.35 [1.40–8.03]).

**Conclusion:**

Severity and duration of food insecurity are important factors in understanding the relationship between food insecurity and HIV outcomes. Our findings highlight the importance of nutritional status, socioeconomic opportunity, and self-stigmatization in the complex pathway between food insecurity and HIV outcomes. Interdisciplinary, multisectoral efforts are needed to develop and implement effective interventions to address food insecurity among people living with HIV.

**Supplementary Information:**

The online version contains supplementary material available at 10.1186/s12889-021-10444-1.

## Background

Food security exists when people have consistent physical, social and economic access to sufficient, safe and nutritious food that meets their dietary needs and food preferences for an active and healthy life [1]. According to the United Nations Food and Agriculture Program, food insecurity is increasing globally, driven primarily by climate change, conflict, economic downturns, and more recently, the COVID-19 pandemic [1].

Sub-Saharan Africa (SSA) is disproportionately affected by both food insecurity and HIV. It is home to nearly a third of the 2 billion people worldwide who are moderately or severely food insecure and two-thirds of the 38 million people living with HIV (PLHIV) [1, 2]. Although PLHIV are particularly vulnerable to the negative consequences of food insecurity [3–8], prospective longitudinal studies to evaluate the associations between food insecurity and HIV outcomes in SSA are limited. Findings from predominantly cross-sectional studies have shown that food insecurity is associated with poor adherence to antiretroviral therapy (ART) among PLHIV in SSA [9–19] and one study conducted in SSA reported an association between food insecurity and lower CD4 cell counts [12, 20]. To our knowledge there has been only one prospective, longitudinal study to determine the association between food insecurity and virologic failure in SSA [12] and there have been no longitudinal studies to evaluate the association between food insecurity and mortality or loss to follow-up (LTFU) among PLHIV in SSA.

Our previous work has shown that the majority of PLHIV in Senegal, West Africa are food insecure [21–23]. Here we report the results of a prospective, longitudinal study to determine the impact of food insecurity on HIV outcomes in Senegal. Understanding the impact of food insecurity on HIV outcomes, and elucidating the mechanisms by which food insecurity impacts PLHIV in SSA, is critical for the development and implementation of effective, evidence-based interventions to address food insecurity and improve the HIV care cascade.

## Methods

All HIV-infected individuals presenting for care and initiation of ART through the Senegalese National AIDS program (ISAARV) at the Clinique des Maladies Infectieuses, Centre Hospitalier National Universitaire de Fann, located in Dakar, and the Centre de Santé de Ziguinchor, located in the Casamance Region, were eligible for enrollment. Informed consent was conducted by the social worker. For participants < 18 years of age, consent was obtained from their legal guardian. Study procedures were approved by the University of Washington Institutional Review Board and the Senegal Comité National d’Ethique pour la Recherche en Santé.

Enrollment took place from April 2017 to April 2018. Data were collected using semi-structured interviews, clinical evaluations, laboratory analyses, and chart review at enrollment, month 6 (M6) and month 12 (M12) (Supplementary file 1). Food insecurity was determined using the Household Food Insecurity Access Scale (HFIAS) [24]. Not food insecure was defined as a HFIAS score of 1 on a scale of 1–4, food insecure was defined as a HFIAS score of 2–4, mildly food insecure was defined as a HFIAS score of 2, moderately food insecure was defined as a HFIAS score of 3, and severely food insecure was defined as a HFIAS score of 4.

HIV-1, HIV-2, and dually infected individuals were enrolled. HIV type was determined using SD Bioline HIV-1/2 3.0 (Alere). Clinical evaluations were performed to determine WHO clinical stage [25]. Nutritional status was determined using body mass index (BMI) and mid-upper arm circumference (MUAC). Undernourished was defined as a BMI < 18.5 kg/m^2^ for non-pregnant participants ≥18 years of age or BMI for age < − 2 z-scores below the median of the WHO Child Growth Standard for participants < 18 years of age [26]. For pregnant women, undernourished was defined as MUAC < 230 mm [27]. For the purposes of this study, the terms undernourishment and undernutrition were used interchangeably. CD4 cell count was measured at enrollment, M6, and M12 using the BD FACSCount system (Becton Dickinson) or the PIMA analyser (Alere).

ART adherence was measured at M6 and M12. Participants were asked by the physician how many times in the past 7 days they failed to take one or more doses of ART, how many times in the past 7 days they failed to take all of their doses of ART, how many times in the past 4 weeks they failed to take one or more doses of ART, and how many times in the past 4 weeks they failed to take all of their doses of ART. They were also asked by both the social worker and the physician if they were always adherent to their ART, not always adherent to their ART, or not adherent to their ART. Those who responded that they were not adherent or not always adherent were classified as poorly adherent. Because more individuals reported poor adherence to ART when asked by the social worker and because this was the only adherence measure that was predictive of virologic failure, adherence as determined by the social worker questionnaire was used for subsequent analysis.

HIV outcomes were determined at the end of the 12 month follow-up period. HIV-1 and HIV-2 plasma RNA viral loads were measured using RealTime HIV-1 and HIV-2 Abbott m2000 platform assays. Virologic failure was defined as > 1000 copies/mL for HIV-1 and > 250 copies/mL for HIV-2. Medical records and family report were used to ascertain mortality. Patients who had no contact with the clinic for > 6 months [28, 29] and could not be traced by phone call or home visit were considered lost to follow-up.

Participants who were retained in care at the end of the 12 month follow-up period were categorized into four main categories according to longitudinal food insecurity status: not severely food insecure at enrollment, severely food insecure at enrollment only, severely food insecure at M0 and M6, and severely food insecure at M0, M6, and M12. This last category is referred to as “persistent severe food insecurity”. Two separate categories included those who were not severely food insecure at enrollment but were severely food insecure at both M6 and M12, and those who were severely food insecure at enrollment and M12 but not M6. Individuals who were not severely food insecure at enrollment but were severely food insecure at one of two follow-up visits were included in the category, not severely food insecure at enrollment. Individuals who were severely food insecure at enrollment but were missing follow-up data for M6 or M12 were classified as missing data and were not included in any of the above categories.

Data were analyzed using SPSS Statistics 26. Descriptive analysis was performed for all variables. Chi-square and Fisher’s Exact tests were used to identify differences in outcomes between individuals who were severely food insecure at enrollment versus those who were not severely food insecure, and between individuals who were undernourished at enrollment versus those who were not undernourished. Logistic regressions were used to identify predictors of HIV outcomes. Missing data were excluded from analysis. *P*-values < 0.05 were considered significant.

## Results

A total of 207 participants were enrolled, 84 (41%) were enrolled in Dakar and 123 (59%) were enrolled in Ziguinchor (Table 1). The majority (70%) were female. The median age was 37 years (IQR 31–46). Approximately 61% of participants were 26–45 years of age, 13% were ≤ 25 years of age, including 6 participants < 18 years of age, and 25% were ≥ 46 years of age. The majority (87%) were infected with HIV-1, 11% were infected with HIV-2, and 2% were dually infected with both HIV-1 and HIV-2. The median CD4 cell count at enrollment was 181 cells/mm^3^ (IQR 63–382), 53% of participants had a baseline CD4 cell count < 200 cells/mm^3^, and 53% had WHO clinical stage 3 or 4 disease.

**Table 1:** Characteristics of 207 HIV-infected individuals presenting for initiation of ART in Senegal

Total number of participants, ***N*** = 207	n	%
**Enrollment site**		
Dakar	84	40.6
Ziguinchor	123	59.4
**Female**	144	69.6
**Age, median years (IQR)**	37	31–46
**Age, years**		
≤ 25	27	13.4
26–35	61	30.3
36–45	62	30.8
≥ 46	51	25.4
**HIV type**		
HIV-1	180	87.0
HIV-2	22	10.6
HIV-1 and HIV-2	5	2.4
**Baseline CD4 cell count**^**a**^**, median (IQR)**	181	63–382
**Baseline CD4 cell count**^**a**^		
0–199	103	53.4
200–499	57	29.5
≥ 500	33	17.1
**WHO clinical stage 3 or 4**	87	52.7
**Food insecure at baseline**^**b**^	137	68.8
Mildly food insecure	16	8.0
Moderately food insecure	63	31.7
Severely food insecure	58	29.1
**Baseline BMI**^**c**^**, median (IQR)**	19.60	16.65–22.98
**Baseline BMI**^**c**^		
< 18.50	71	40.6
18.50–24.99	81	46.3
25.00–29.99	16	9.1
≥ 30.00	7	4.0
**Undernourished**^**d**^	76	38.4
**Highest educational level obtained**		
No formal education	56	32.2
Any primary school	68	39.1
Any secondary school	50	28.7
**Unemployed**	160	86.5
**Practice agriculture**	46	23.1
**Own livestock**	79	39.5
**Married**	107	55.2
**Number of children, median (IQR; range)**	2	1–4; 0–10
**Household size, median (IQR)**	7	5–12
**Household daily food expenditure per person, USD median (IQR)**	$0.58	$0.27 - $0.97
**Household daily food expenditure per person, USD**		
< $0.25	31	21.7
$0.25 - $0.49	30	21.0
$0.50 - $0.99	48	33.6
≥ $1.00	34	23.8
**Transportation time to HIV clinic, median minutes (IQR)**	90	30–240
**Transportation time, minutes**		
≤ 60	87	44.6
61–120	36	18.5
> 120	72	36.9
**Transportation cost to HIV clinic, USD median (IQR)**	$1.74	$0.70 - $1.83
**Transportation cost, USD**		
< $1.00	67	35.4
$1.00 - $2.49	86	45.5
≥ $2.50	36	19.0
**Have not disclosed HIV status to anyone**	74	46.5

^a^Missing data for 14 participants; ^b^Missing data for 8 participants; ^c^BMI among non-pregnant participants ≥18 years of age, *N* = 175; ^d^Missing data for 9 participants

**Fig. 1:**
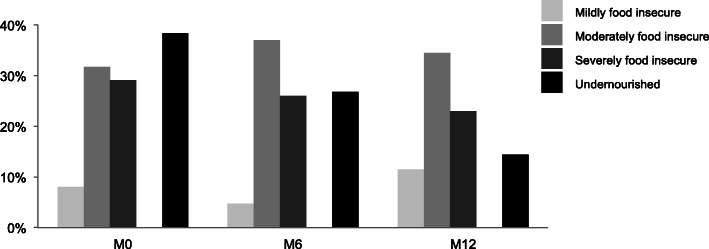
Food insecurity^a^ and nutritional status^b^ at enrollment, 6 months and 12 months among HIV-infected individuals in Senegal. ^a^Among 199 participants at M0, 127 at M6, and 113 at M12; ^b^Among 198 participants at M0, 123 at M6, and 104 at M12

The majority (69%) of participants were food insecure at enrollment; 8% were mildly food insecure, 32% were moderately food insecure, and 29% were severely food insecure (Fig. 1). The median baseline BMI among non-pregnant participants ≥18 years of age was 19.6 (IQR 16.7–23.0) and 38% of participants were undernourished at enrollment. Neither baseline BMI nor proportion undernourished at baseline were significantly different among those who were food insecure versus those who were not food insecure at enrollment (BMI:19.75 versus 18.87, *p* = 0.76; undernourished: 35% versus 44%, *p* = 0.26).

Nearly a third (32%) of participants had no formal education, 39% had attended some primary school, and 29% had attended secondary school. The majority (87%) had no formal employment. Approximately 23% of participants practiced agriculture, 40% owned livestock, and 4% practiced fishing. Among those who practiced agriculture, 41% raised crops for food and 59% raised crops for both food and income. Crops included rice, peanuts, millet, corn, beans, vegetables, and bananas. Among those who owned livestock, 44% raised livestock for food, 9% raised livestock for income, 40% raised livestock for both food and income, and 7% raised livestock for neither food nor income. Livestock included chickens, ducks, pigeons, sheep, goats, pigs, and cows.

The majority (55%) of participants were married, of which 71% were monogamous and 29% were polygamous. The median number of children was 2 (IQR 1–4; range 0–10). The median household size was 7 (IQR 5–12). The median household daily food expenditure per person was $0.58 (IQR $0.27–$0.97), 22% of participants spent less than $0.25 per day on food per person and 76% of participants spent less than $1.00 per day on food per person. Food expenditure was lower among those who practiced agriculture ($0.35) or owned livestock ($0.36) compared to those who did not practice agriculture ($0.62, *p* < 0.01) or own livestock ($0.70, *p* < 0.01). The median round trip transportation time to the HIV clinic was 90 min (IQR 30–240). Transportation time did not differ among those who practiced agriculture or owned livestock compared to those who did not. The median cost of transportation to the HIV clinic was $1.74 (IQR $0.70–$1.83), which was three times the median daily food expenditure per person.

At the time of enrollment, 47% of participants had not told anyone that they were HIV-positive, of which 31% reported that they had not disclosed their status due to fear of stigmatization or because they were ashamed. At M12, 43% had not shared their status, of which 32% reported that they had not disclosed their status due to fear of stigmatization or because they were ashamed. Non-disclosure of HIV-status due to self-stigmatization, which included fear of stigmatization and being ashamed, was more common among those who were food insecure versus not foot insecure (48% versus 8%, *p* = 0.02). The median household daily food expenditure per person was lower among those who did not share their status compared to those who did ($0.50 [$0.28 - $0.70] versus $0.73 [$0.39 - $1.30], *p* = 0.048). Nutritional status did not differ between groups.

Following 12 months of ART, 131 participants (63%) were retained in care, 27 (13%) were alive but not retained in care at the enrollment clinic, 19 (9%) were LTFU, and 30 (15%) had died. M12 viral loads were available for 107 participants, of which 16 (15%) experienced VF (Table 2). Poor adherence to ART was reported by 8% of participants and 26% had CD4 cell counts < 200.

**Table 2:** HIV outcomes at 12 months among HIV-infected individuals in Senegal according to baseline food insecurity and nutritional status

	Among all participants	Food insecurity, ***N*** = 199^**a**^			Nutritional status, ***N*** = 198^**b**^		
		Not severe at M0, ***N*** = 141 n (%)	Severe at M0, ***N*** = 58 n (%)	***p***-value	Not undernourished at M0, ***N*** = 122 n (%)	Undernourished at M0, ***N*** = 76 n (%)	***p***-value
**VF**^**c**^	16 (15.0)	10 (13.3)	6 (19.4)	0.55	**6 (9.0)**	**10 (25.0)**	**0.02**
**Lost to follow-up**	19 (9.2)	**7 (5.0)**	**9 (15.5)**	**0.02**	12 (9.8)	5 (6.6)	0.43
**Died**	30 (14.5)	20 (14.2)	7 (12.1)	0.69	**9 (7.4)**	**16 (21.1)**	**0.01**
**Poor adherence**^**d**^	9 (8.0)	5 (6.3)	4 (12.9)	0.26	7 (9.7)	2 (5.0)	0.49
**CD4 count < 200 at M12**^**e**^	23 (25.6)	18 (28.1)	4 (16.0)	0.23	**9 (15.5)**	**14 (43.8)**	**< 0.01**

^a^Missing data for 8 participants; ^b^Missing data for 9 participants; ^c^M12 viral loads available for 107 participants; ^d^M12 adherence available for 112 participants; ^e^M12 CD4 counts available for 90 participants

Those with severe food insecurity at baseline were more likely to be LTFU than those without severe food insecurity at baseline (16% vs. 5%; *p* = 0.02). VF, death, poor adherence to ART, and CD4 count < 200 at M12 were similar in those with and without severe food insecurity at baseline.

Those who were undernourished at baseline were more likely to have VF (25% vs. 9%; *p* = 0.02), more likely to die (21% vs. 7%, *p* = 0.01; OR 3.35 [95% CI 1.40–8.03], *p* = 0.01), and more likely to have a CD4 count < 200 at M12 (44% vs. 16%; *p* < 0.01) than those who were not undernourished at baseline. Loss to follow-up and poor adherence to ART were similar among those who were undernourished versus not undernourished at baseline.

Six months after initiating ART, 68% of participants were food insecure, 37% were moderately food insecure, and 26% were severely food insecure. Twelve months after initiating ART, 69% of participants were food insecure, 35% were moderately food insecure, and 23% were severely food insecure. The median BMI at M6 among non-pregnant participants ≥18 years of age was 20.71 (IQR 18.39–24.94) and 27% were undernourished. The median BMI at M12 among non-pregnant participants ≥18 years of age was 21.51 (IQR 19.60–24.90) and 14% were undernourished.

The median BMI at both M6 and M12 was lower among those who were food insecure at M6 or M12 compared to those who were not food insecure at M6 or M12 (M6 BMI: 20.47 versus 23.51, *p* = 0.03; M12 BMI: 20.61 versus 23.51, *p* = 0.04). The proportion undernourished at M6 did not differ significantly among those who were food insecure at M6 versus those who were not food insecure at M6 (30% versus 20%, *p* = 0.23), nor did proportion undernourished at M12 differ among those who were food insecure at M12 versus those who were not food insecure at M12 (16% versus 13%, *p* = 0.77). However, those who were food insecure at M12 were more likely to have been undernourished at M0 compared to those who were not food insecure at M12 (45% versus 20%, *p* = 0.01).

Among the 118 participants who had follow-up food insecurity scores, 84 (71%) were not severely food insecure at enrollment. Among those who were severely food insecure at enrollment and had follow-up food insecurity scores for M6 and M12, 12 (10%) were severely food insecure at enrollment but not M6 or M12, 5 (4%) were severely food insecure at enrollment and M6 but not M12, and 8 (7%) were severely food insecure at enrollment, M6, and M12 (referred to as “persistent severe food insecurity”). Four individuals (3%) were not severely food insecure at enrollment but were severely food insecure at M6 and M12, and 5 (4%) were severely food insecure at enrollment and M12 but not M6.

**Fig. 2:**
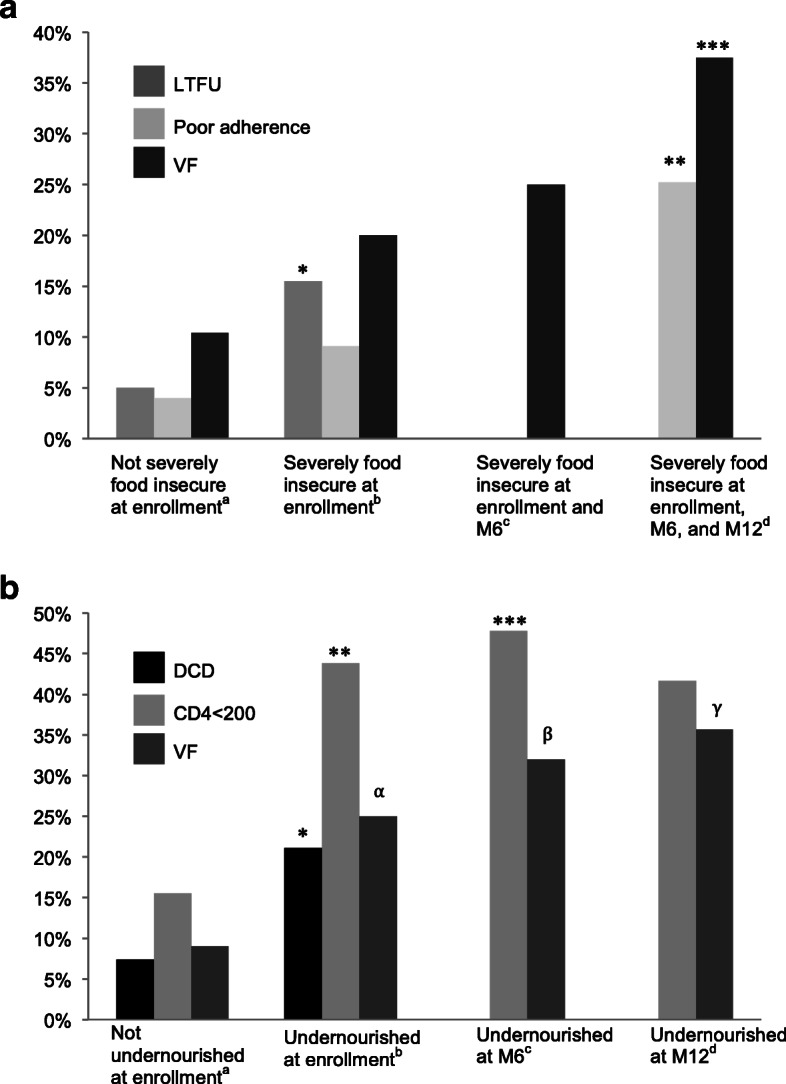
**a** Percent of HIV-infected individuals who were lost to follow-up (LTFU) within 12 months of starting ART, or reported poor adherence to ART, or experienced virologic failure (VF) at 12 months of ART according to food insecurity status. *OR for LTFU 3.13 [1.08–9.06] compared to not severely food insecure at enrollment. **OR for poor adherence 8.00 [1.11–57.57] compared to not severely food insecure at enrollment. ***OR for VF 5.14 [1.01–26.29] compared to not severely food insecure at enrollment. ^a^*N* = 141 at baseline, 84 among those retained in care; ^b^*N* = 58 at baseline,12 among those retained in care; ^c^*N* = 5; ^d^*N* = 8. **b** Percent of HIV-infected individuals who died within 12 months of initiating ART, or had CD4 cell counts < 200 following 12 months of ART, or experienced virologic failure (VF) at 12 months of ART according to nutritional status. *OR for DCD 3.35 [1.40–8.03] compared to not undernourished at enrollment. **OR for CD4 < 200 4.24 [1.56–11.47] compared to not undernourished at enrollment; ***OR for CD4 < 200: 5.84 [1.93–17.67] compared to not undernourished at M6. ^α^OR for VF 3.39 [1.13–10.21] compared to not undernourished at enrollment; ^β^OR for VF 7.41 [1.99–27.59] compared to not undernourished at M6; ^γ^OR for VF 4.14 [1.13–15.11] compared to not undernourished at M12. ^a^*N* = 122 at baseline, 87 among those retained in care; ^b^*N* = 76 at baseline, 44 among those retained in care; ^c^*N* = 31; ^d^*N* = 15

Among those retained in care who had follow-up food insecurity scores, the frequency of poor HIV outcomes at 12 months increased with increased duration of severe food insecurity (Fig. 2a). Poor adherence to ART at M12 was reported by 4% of those who were not severely food insecure at enrollment, 9% who were severely food insecure at enrollment only, 0% who were severely food insecure at M0 and M6 only, and 25% who were severely food insecure at M0, M6, and M12. Those who were severely food insecure at M0, M6, and M12 were more likely to report poor adherence compared to those who were not severely food insecure at enrollment (OR 8.00 [1.11–57.57]. Virologic failure (VF) occurred in 10% of participants who were not severely food insecure at enrollment, 20% who were severely food insecure at enrollment only, 25% who were severely food insecure at M0 and M6 only, and 38% who were severely food insecure at M0, M6, and M12.

Among those retained in care, 87 (66%) were not undernourished at enrollment, 44 (34%) were undernourished at enrollment, 31 (27%) were undernourished at M6, and 15 (14%) were undernourished at M12. Among those retained in care, a CD4 cell count < 200 at M12 occurred in 16% of participants who were not undernourished at enrollment, 44% who were undernourished at enrollment, 48% who were undernourished at M6, and 42% who were undernourished at M12 (Fig. 2b).

Those who were undernourished at M0 or M6 were more likely to have M12 CD4 cell counts < 200 compared to those who were not undernourished at M0 or M6 (OR for M0: 4.24 [1.56–11.47] compared to not undernourished at enrollment; OR for M6: 5.84 [1.93–17.67] compared to not undernourished at M6; OR for M12: 3.16 [0.85–11.67] compared to not undernourished at M12). Among those retained in care, VF occurred in 9% of participants who were not undernourished at enrollment, 25% who were undernourished at enrollment, 32% who were undernourished at M6, and 36% who were undernourished at M12.

The strongest predictors of loss to follow-up were severe food insecurity at enrollment (OR 3.13 [1.08–9.06]) and daily food expenditure per person <$0.25 (OR 4.32 [1.07–17.42]) (Table 3).

**Table 3:** Logistic regressions showing predictors of loss to follow-up within 12 months of starting ART among HIV-infected individuals in Senegal

	**Simple regressions**			
	**OR**	**95% CI**		***p*-value**
**Ziguinchor site** (ref. Dakar)	**4.14**	**1.16**	**14.76**	**0.03**
**Female** (ref. male)	2.26	0.63	8.12	0.21
**Age** (ref. 26–35)				
≤ 25	2.48	0.46	13.27	0.29
36–45	2.89	0.70	11.86	0.14
≥ 46	2.81	0.66	11.97	0.16
**Education** (ref. no education)				
Any primary school	0.44	0.13	1.45	0.18
Any secondary school	0.23	0.05	1.16	0.08
**Transportation cost** (ref. <$1.00)				
$1.00–$2.49	0.50	0.14	1.80	0.29
≥ $2.50	1.21	0.33	4.48	0.78
**For every $0.10 increase in transportation cost**	1.05	0.44	2.52	0.91
**Transportation time** (ref ≤60 min)				
61–120	1.17	0.27	4.98	0.84
> 120	1.08	0.31	3.73	0.90
**Food insecurity at M0**				
Any food insecurity at M0	1.98	0.54	7.28	0.30
Severe food insecurity at M0	**3.49**	**1.22**	**9.96**	**0.02**
**Undernourished at M0**	0.77	0.26	2.28	0.63
**Food expenditure per person < $0.25**	**6.21**	**1.61**	**23.89**	**0.01**
**For every $0.10 decrease in food expenditure**	**1.42**	**1.05**	**1.91**	**0.02**
**Practice agriculture**	1.11	0.34	3.66	0.86
**Own livestock**	2.02	0.72	5.72	0.18
**Had not disclosed HIV status to anyone at M0**	2.65	0.66	10.71	0.17
	**Multiple regressions**			
	**OR**	**95% CI**		***p*-value**
**Ziguinchor site** (ref. Dakar)	2.94	0.79	10.90	0.11
**Severe food insecurity at M0**	**3.13**	**1.08**	**9.06**	**0.04**
	**OR**	**95% CI**		***p*-value**
**Ziguinchor site** (ref. Dakar)	4.55	0.52	39.72	0.17
**Food expenditure per person < $0.25**	**4.32**	**1.07**	**17.42**	**0.04**
	**OR**	**95% CI**		***p*-value**
**Ziguinchor site** (ref. Dakar)	3.09	0.35	26.98	0.31
**For every $0.10 decrease in food expenditure**	1.36	1.00	1.86	0.05

The strongest predictors of VF were severe food insecurity at M0, M6, and M12, referred to as “persistent severe food insecurity” (OR 5.14 [1.01–26.29]), undernourished at enrollment or any follow-up visit (OR for M0: 3.39 [1.13–10.21] compared to not undernourished at enrollment; OR for M6: 7.41 [1.99–27.59] compared to not undernourished at M6; OR for M12: 4.14 [1.13–15.11] compared to not undernourished at M12) and the practice of agriculture (OR 4.29 [1.41–13.07]) (Table 4).

**Table 4:** Logistic regressions showing predictors of virologic failure following 12 months of ART among HIV-infected individuals in Senegal

	Simple regressions			
	OR	95% CI		*p*-value
**Ziguinchor site** (ref. Dakar)	2.67	0.89	7.99	0.08
**Female** (ref. male)	0.60	0.20	1.82	0.36
**Age** (ref. 26–35)				
≤ 25	2.05	0.39	10.64	0.40
36–45	0.23	0.02	2.15	0.20
≥ 46	3.50	0.88	13.86	0.07
**Education** (ref. no education)				
Any primary school	0.68	0.15	3.01	0.61
Any secondary school	1.14	0.27	4.83	0.86
**Unemployed**	1.07	0.21	5.34	0.94
**Transportation cost** (ref. <$1.00)				
$1.00–$2.49	0.92	0.27	3.12	0.90
≥ $2.50	0.92	0.20	4.11	0.91
**For every $0.10 increase in transportation cost**	1.24	0.59	2.59	0.57
**Transportation time** (ref ≤60 min)				
61–120	0.20	0.02	1.74	0.15
> 120	0.41	0.13	1.35	0.14
**Any food insecurity at M0**	1.86	0.49	7.05	0.36
**Severe food insecurity** (ref. Not severe at M0)				
Severe at M0	2.14	0.38	12.16	0.39
Severe at M0 and M6	2.86	0.26	31.33	0.39
Severe at M0, M6, and M12	**5.14**	**1.01**	**26.29**	**0.049**
**Nutritional status**				
Undernourished at M0	**3.39**	**1.13**	**10.21**	**0.03**
Undernourished at M6	**7.41**	**1.99**	**27.59**	**< 0.01**
Undernourished at M12	**4.14**	**1.13**	**15.11**	**0.03**
**Food expenditure per person < $0.25**	1.36	0.26	7.21	0.72
**For every $0.10 decrease in food expenditure**	1.05	0.95	1.17	0.32
**Practice agriculture**	**4.29**	**1.41**	**13.07**	**0.01**
**Own livestock**	1.53	0.53	4.44	0.44
**Had not disclosed HIV status to anyone at M0**	0.48	0.12	1.91	0.30
**Had not disclosed HIV status to anyone at M12**	0.92	0.28	2.99	0.89

## Discussion

In this prospective longitudinal study conducted in Senegal, West Africa, we followed HIV-positive individuals during the first 12 months of ART to evaluate the impact of food insecurity on HIV outcomes. We found that severe food insecurity is a strong predictor of LTFU and persistent severe food insecurity is associated with VF and poor adherence to ART.

Food insecurity and HIV are tightly linked in a bidirectional relationship [3] whereby each condition is exacerbated by the other. Individuals with poorly controlled HIV are at greater risk of food insecurity, and individuals who are food insecure may be at increased risk of HIV infection and poor HIV outcomes [3–21, 30–42]. HIV can contribute to food insecurity as individuals with poorly controlled disease may have decreased physical, social, and economic productivity, compounded by a degradation of household resources due to health related expenses [4, 43–48]. These conditions can lead to weaknesses in the four dimensions of food security, namely, food availability, access, utilization, and stability over time [1]. While the focus of this study was the impact of food insecurity on HIV outcomes, it is noteworthy that neither the prevalence nor severity of food insecurity decreased following 12 months of ART. Further studies, including rigorous qualitative studies, are indicated to identify and characterize the factors that drive and maintain food insecurity in this population.

**Fig. 3:**
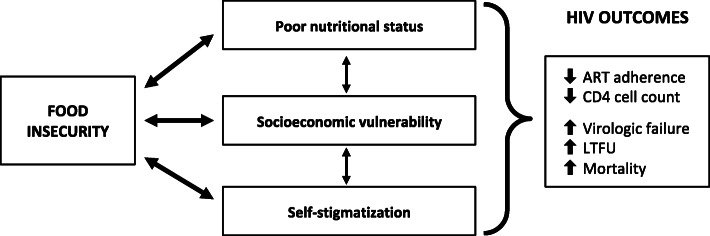
Theoretical framework showing pathways by which food insecurity may impact HIV outcomes

We hypothesized that food insecurity would contribute to poor HIV outcomes through three interrelated pathways, mediated by poor nutritional status, socioeconomic vulnerability, and self-stigmatization (Fig. 3).

### Poor nutritional status

A comprehensive understanding of the multidirectional relationships between food insecurity, nutritional status, and HIV requires consideration of the severity and duration of food insecurity, nutritional status over time, and the extent of control over HIV-infection. Furthermore, attention to the measure of food insecurity utilized is important. We used the HFIAS, which focuses on the dimension of food access [24]. We measured food insecurity at three time points during the first 12 months of ART in an effort to capture the dimension of stability over time.

We found that poor nutritional status was associated with poor immunologic recovery, VF, and death. Although food insecurity and nutritional status were not associated at baseline, those who were undernourished at baseline were more likely to be food insecure following 12 months of ART and persistent food insecurity was associated with lower BMI. These findings are consistent with the bidirectional relationship between food insecurity and nutritional status. Weaknesses in any one of the four dimensions of food insecurity can contribute to poor nutritional status. Similarly, poor nutritional status can exacerbate food insecurity. Individuals with poor nutritional status may be unable to participate in activities that ensure access to sufficient, safe and nutritious food, and undernutrition and its associated conditions can impede the body’s ability to adequately utilize nutrients.

While food insecurity can be an important factor contributing to undernutrition, undernutrition can be caused by other factors. As such, the impact of poor nutritional status on HIV outcomes can result from pathways that are independent of food insecurity [4, 49]. Undernutrition, which is defined as, “the outcome of poor nutritional intake in terms of quantity and/or quality, and/or poor absorption and/or poor biological use of nutrients consumed,” [1] is a direct cause of poor immune function and is associated with malabsorption, which can diminish the effects of ART [50–53]. Similarly, poorly controlled HIV can contribute to undernutrition, as HIV infection and associated inflammation can impair appetite, digestion, metabolic function, and absorption and utilization of nutrients [4, 49–51, 54].

### Socioeconomic vulnerability

Socioeconomic vulnerability is a fundamental factor driving the complex interactions in our theoretical framework, as individuals who are socioeconomically vulnerable are at increased risk of food insecurity, poor nutritional status, stigmatization, and poor health outcomes. In this study, individuals with lower daily food expenditures were more likely to be LTFU. In a setting where the cost of transportation to the HIV clinic is three times the daily food expenditure, individuals who are socioeconomically vulnerable are faced with conflicting demands on insufficient resources. Confronted by the decision to purchase food for the household or pay for transportation to clinic, some individuals may prioritize food expenditure over transportation expenditure, resulting in greater LTFU. Similarly, socioeconomically vulnerable individuals may be faced with conflicting demands on time. They may prioritize time spent earning income to pay for food rather than spending a substantial amount of time traveling to clinic and waiting for their clinic appointment. Findings from qualitative studies conducted among food insecure PLHIV in SSA have described similar conflicting demands on resources and time; the cost of transportation to clinic in the setting of limited resources for household food, and lost wages due to travel and clinic wait times, are reported barriers to HIV treatment and ART adherence [37–40].

Conflicting demands on time may also contribute to the association between the practice of agriculture and VF, as socioeconomically vulnerable farmers may prioritize time spent on agricultural needs rather than traveling to clinic or returning home to take their ART during intensive planting and harvesting periods. In previous studies conducted among PLHIV in SSA, agricultural work responsibilities have been reported as a barrier to ART adherence [36]. The association between the practice of agriculture and VF is an important finding that warrants further investigation and identifies farmers as a vulnerable population in Senegal, at high risk of poor HIV outcomes.

### Self-stigmatization

We found that non-disclosure of HIV status, even among married participants, was common among participants in this study. Non-disclosure of HIV status is considered a “proximate consequence” of self-stigmatization [55, 56]. Self-stigmatization includes enacted stigma, anticipated stigma and internalized stigma. Internalized stigma is the development of self-defacing beliefs and perceptions about oneself resulting from the acceptance of the negative attitudes of others as valid [55–59]. Anticipated stigma can manifest as fear of disclosing one’s HIV status and internalized stigma can lead to feelings of shame. In this study, food expenditure was lower among those who did not disclose their status and food insecurity was associated with non-disclosure of HIV-status due to fear of stigmatization and feelings of shame.

Multiple prior studies have shown that non-disclosure of HIV status is associated with poor HIV outcomes, including decreased adherence to ART and increased risk of HIV transmission [55, 60, 61]. Furthermore, self-stigmatization can lead to decreased social engagement, depression, and decreased quality of life [55–61]. In this study, self-stigmatization was determined using open-ended interview questions related to non-disclosure of HIV status. Future studies employing a standardized stigma scale may be more effective in capturing and characterizing the relationship between self-stigmatization and HIV outcomes in this community.

The primary limitations of our study were small sample size and incomplete data. HIV-1 viral loads were not available for 14 of the 115 participants with HIV-1 who were retained in care and 10 of the 12 participants with HIV-2 who were retained in care. We evaluated only two HIV clinics in Senegal, which could limit the generalizability of our findings. Although self-reported adherence was associated with virologic outcomes, measurement of ART levels in patient blood or urine would have provided a more reliable measure. Finally, our findings would have been strengthened by the use of a validated quantitative measure of stigma.

## Conclusion

Prospective longitudinal studies are necessary to accurately delineate the impact of food insecurity on HIV outcomes. In this study, severity and duration of food insecurity were important factors in understanding the relationship between food insecurity and HIV. We found that individuals with severe or persistent food insecurity were at increased risk of poor outcomes. Furthermore, our findings highlight the importance of nutritional status, socioeconomic opportunity, and self-stigmatization in the complex pathway between food insecurity and HIV outcomes. Interdisciplinary, multisectoral efforts are needed to develop and implement effective interventions to address food insecurity among PLHIV in SSA.

## Supplementary Information

**Additional file 1.** English translation of study questionnaire

## Data Availability

The datasets used and/or analyzed during the current study are available from the corresponding author on reasonable request.

## Abbreviations

ART: Antiretroviral therapy
BMI: Body mass index
DCD: Deceased
HFIAS: Household Food Insecurity Access Scale
IQR: Interquartile range
ISAARV: Initiative sénégalaise d’accès aux antirétroviraux
LTFU: Loss to follow-up
M0: Month 0
M6: Month 6
M12: Month 12
MUAC: Mid-upper arm circumference
OR: Odds ratio
PLHIV: People living with HIV
SSA: Sub-Saharan Africa
VF: Virologic failure
