# Supplementary material for: The impact of food insecurity on HIV outcomes in Senegal, West Africa: a prospective longitudinal study
Source: BMC Public Health. 2021 Mar 6;21:451. doi: 10.1186/s12889-021-10444-1 (PMC7936446; doi:10.1186/s12889-021-10444-1)
Supplement: Supplementary file 1 — Additional file 1. English translation of study questionnaire [file 12889_2021_10444_MOESM1_ESM.pdf]

The impact of food insecurity on HIV outcomes in Senegal, West Africa: A prospective longitudinal study

English translation of study questionnaire

**ENROLLMENT**
**PART 1**
**Participant age:**
**Participant sex:**
**Where were you born (country, region, department)?**
**Where do you live currently (country, region, department)?**
**How long does it take for you to travel from home to the clinic?**
**What means of transportation do you use to get to the clinic?**
**What is the round trip cost of transportation to the clinic?**
**What is your marital status:**
**Have you shared your HIV status with anyone?**
If YES, with who?
If NO, why haven't you shared your status?
**What is your highest educational level?** No school Primary school Secondary school University
**Can you read and write?**
**Do you know how to do arithmetic?**
**Are you employed?**
If you were employed previously, why are you no longer employed?<br>If you are no longer working because of HIV/AIDS, explain:
**What is your profession?**
**How many children to you have?**
Have your children been tested for HIV? Yes No Don't know
**Number of people in the household:**
**Number of people in the household <5 years of age:**
**Number of people in the household ≥18 years of age:**
**Number of people in household who are employed:**
**Do you receive financial support in the form of donations or transfers?**
If YES, from whom, the amount and the frequency:
**Do you provide financial support in the form of donations or transfers?**
If YES, to whom, the amount and the frequency:
**Do you have a personal cell phone?**
If YES, is there any credit?
**Is there electricity in the household?**
**Is there tap water in the household?**
**Type of household toilet:**
**Type of household floor:**
**Do you have or have you ever had livestock?**
If YES, specify type of animal and number of each:
If you previously had livestock, please explain why you no longer have livestock:
Do you eat your livestock or their products (eggs, milk, etc.)?
Do you sell your livestock or their products (eggs, milk, etc.)?
**Do you or have you ever practiced agriculture or gardening?**

If YES, what are you cultivating?
If you previously practiced agriculture or gardening, please explain why you no longer practice agriculture or gardening:
Do you eat the products you cultivate?
Do you sell the products you cultivate?
**Do you practice fishing?**
If YES, do you eat what you catch?
Do you sell what you catch?
**Who prepares the meals in your household?**
**Who buys food for your household?**
**How much is spent per day on food in your household?**
**How many people contribute money for food for the household?**
**Have you ever begged for food?**
**Have you ever traded for food?**
**Have you ever received donated food?**
**Have you ever received food aid?**
If YES, please describe the type of aid, when it was received, and from which organization:

PART 2			
**Type of HIV:**			
**Date of HIV diagnosis:**			
**Reason for screening:**			
**WHO stage:**	**Date:**		
**CD4 nadir:**	**Date:**		
**Most recent CD4:**	**Date:**		
**Prior TB testing:**	**Date:**		
**Have you had a cough in the last 24hrs?**			
**Have you had a fever in the last 24hrs?**			
**Have you had diarrhea in the last 24hrs?**			
**Have you experienced any challenges getting tested for HIV?**			
If YES, please explain:			
**Have you experienced any challenges getting treatment for HIV?**			
If YES, please explain:			
**Anticipated ART start date:**			
**Anticipated ART regimen:**			
If ART dispensed today, number of doses dispensed:			
Number of tablets per day:	Morning:	Afternoon:	Evening:
**Have you received ART previously?**			
If YES, between which dates:			
If YES, why did you stop taking ART?			
If YES, what ART did you receive previously?			
**Are you currently taking co-trimoxazole:**			
If YES, when did you start taking co-trimoxazole?			
**List all other medications given to the patient and the date when they were prescribed:**			
**Is the participant currently pregnant?**			
If YES, provide the due date:			
**Is the participant currently breastfeeding?**			
**Does the participant have any other medical problems?**			

If YES, specify:
**Is the participant taking any other drugs?**
If YES, specify:
**When was the last time you visited a doctor ?**
**What was the reason for your last visit to the doctor?**
**Have you visited a traditional practitioner?**
If YES, what was the reason for your last visit to the traditional practitioner?<br>When was the last visit (date)?

**FOLLOW-UP: MONTH 6 MONTH 12**
**PART 1**
**Are you taking ARVs ?** YES, ALWAYS YES, BUT NOT ALWAYS NO<br>If YES, since when?<br>If YES but not always, why?<br>If NO, why not ?
**Did you have any problems getting your ARVs ?**<br>If YES, explain:
**What are the challenges to adherence to ART?**
**Has your marital status changed since the last study visit ?**<br>If YES, how ?
**Have you shared your HIV status?**<br>If YES, with whom?<br>If NO, why haven't you shared your status?
**Have you had any children since the last study visit ?**<br>If YES, how many ?<br>Have your children been tested for HIV? Yes No Don't know
**Are you employed ?**<br>If you were employed previously, why are you no longer employed?<br>If you are no longer working because of HIV/AIDS, explain:
**Profession:**<br>**Salary per month :**<br>**Has your salary changed since the last visit ?**<br>If YES, how?
**Do you have a personal cell phone?**<br>If YES, is there any credit?
**How long does it take for you to travel from home to the clinic?**<br>**What means of transportation do you use to travel to the clinic?**<br>**What is the cost of transportation to the clinic (round trip)?**
**Have you moved since the last study visit ?**<br>If YES:<br>Why did you move?<br>Where do you currently live?<br>Is there electricity in the new household?<br>Is there tap water in the new household?<br>Type of toilet in the new household:<br>Type of floor in the new household:
**Has the number of people in the household changed since the last visit ?**<br>If YES:<br>Number of people in the household <5 years of age:<br>Number of people in the household ≥18 years of age:<br>Number of people in the household employed:
**Do you receive financial support in the form of donations or transfers?**<br>If YES, from whom, the amount and the frequency:
**Do you provide financial support in the form of donations or transfers?**<br>If YES, to whom, the amount and the frequency:

**Do you have or have you ever had livestock?**<br>If YES, specify type of animal and number of each:<br>If you previously had livestock, please explain why you no longer have livestock:<br>Do you eat your livestock or their products (eggs, milk, etc.)?<br>Do you sell your livestock or their products (eggs, milk, etc.)?
**Do you or have you ever practiced agriculture or gardening?**<br>If YES, what are you cultivating?<br>If you previously practiced agriculture or gardening, please explain why you no longer practice agriculture or gardening:<br>Do you eat the products you cultivate?<br>Do you sell the products you cultivate?
**Do you practice fishing?**<br>If YES, do you eat what you catch?<br>Do you sell what you catch?
**Who prepares the meals in your household?**
**Who buys food for your household?**
**How much is spent per day on food in your household?**
**How many people contribute money for food for the household?**
**Since the last visit:**<br>**Have you begged for food?**<br>**Have you traded for food?**<br>**Have you received donated food?**<br>**Have you received food aid?**<br>If YES, when? What kind of aid? From which organization:

**PART 2**
**WHO stage:** 1 2 3 4 **Date:**
**New stage 3 or 4 clinical events since the last visit?** Yes No<br>**If YES, describe:**
**Most recent CD4:** **Date**
**Current ART regimen:**
**Are you taking ARVs ?** YES, ALWAYS YES, BUT NOT ALWAYS NO<br>If YES, since when?<br>If YES but not always, why?<br>If NO, why not?
**How many ARV tablets per day in total?** **Morning:** **Afternoon:** **Evening:**
**Are you taking Co-trimoxazole:**
**In the past 7 days, how many days did you miss taking all of your ARV tablets ?**
**In the past 7 days, how many days did you miss taking ≥1 ARV tablets ?**
**In the past 4 weeks, how many days did you miss taking all of your ARV tablets ?**
**In the past 4 weeks, how many days did you miss taking ≥1 ARV tablets ?**
**Have you had any problems getting your ARVs ?**<br>If YES, explain:
**What are the challenges to adherence to ART?**
**Have you had any interruptions in ART?**<br>If YES, why?
**Has your ART been interrupted due to stock-outs?**
**Have you missed taking medication due to lack of food ?**
**Have you had any side effects that kept you from eating ?**<br>If YES, describe:

**Have you had a cough in the last 24 hours?**
**Have you had a fever in the last 24 hours?**
**Have you had diarrhea in the last 24 hours?**
**List all other medications given to the patient and the date when they were prescribed:**
**Is the participant currently pregnant?**<br>If YES, how many months along in pregnancy:
**Is the participant currently breastfeeding?**
**Do you have any other medical problems?**<br>If YES, explain:
**Are you taking any other medications OR traditional treatments ?**<br>If YES, explain:
**When was the last time you visited a doctor ?** Date :
**What was the reason for your last visit to the doctor?**
**Have you visited a traditional practitioner?**<br>If YES, what was the reason for your last visit to the traditional practitioner?<br>When was the last visit (date)?
**PRESCRIPTIONS**
**Has the participant's ART regimen been changed since the last visit?**<br>If YES**:**<br>How many times?<br>Why?
**List medications prescribed today:**
**Will ART be dispensed today?**<br>If NO:<br>Why not ?<br>When will ART be dispensed ?<br>**If ART will be dispensed today:**<br>How many days of ART dispensed today?<br>Before today, the date of the last ART refill:<br>Number of days since ART last filled:<br>How many days of ART were provided when last filled?
